# Effect of Needle-Tract Bleeding on Pneumothorax and Chest Tube Placement Following CT Guided Core Needle Lung Biopsy

**DOI:** 10.5334/jbsr.1591

**Published:** 2019-04-04

**Authors:** Esra Soylu, Kerem Ozturk, Gokhan Gokalp, Ugur Topal

**Affiliations:** 1 Radiology Clinic, Cekirge State Hospital, Bursa, TR; 2 Department of Radiology, Uludag University Faculty of Medicine, Bursa, TR

**Keywords:** pneumothorax, biopsy, chest tube drainage, pulmonary nodule, computed tomography

## Abstract

**Background:**

Bleeding in the biopsy tract has been studied for its ability to decrease the risk of pneumothorax with indefinite results in the previous studies.

**Purpose:**

To investigate the risk factors for needle-tract bleeding (NTB) and the possible effect of NTB on the pneumothorax and resultant chest tube placement after CT-guided cutting needle biopsy (CT-CNB) of pulmonary lesions.

**Methods:**

Predictive variables for NTB and the effect of NTB on the development of pneumothorax and consequent chest tube placement were retrospectively determined in 416 patients who had undergone an 18-gauge non-coaxial CT-CNB (338 men and 78 women; average age, 59.3 years). Patient-related parameters were age, gender, patient position, and severity of pulmonary emphysema. Lesion-related variables were size, localization, and contour characteristics of the lesion. Procedure-related variables were the presence of atelectasis, pleural tag, and fissure in the needle-tract, length of the aerated lung parenchyma crossed by needle, needle entry angle, number of pleural punctures, the experience of the operator, and procedure duration. All variables were analyzed by x^2^ test and logistic regression analysis.

**Results:**

NTB was demonstrated in 142 of 421 (33.7%) procedures. The predictive variables of NTB were smaller lesion size (p = 0.011) and greater lesion depth (p = 0.002). In patients without emphysema around the lesion, the pneumothorax developed in 44/190 cases (23.1%) without NTB and in 12/95 procedures (12.6%) with NTB (p < 0.001).

**Conclusion:**

NTB may have a preventive effect on pneumothorax development, particularly in the absence of emphysema around the lesion.

## Introduction

Computed tomography (CT)-guided core needle lung biopsy (CT-CNB) is a reliable and safe diagnostic procedure; the most common complication of CT-CNB is pneumothorax [1,2]. Some invasive techniques, such as injection of sealant materials (e.g., autologous blood patch, collagen plugs, fibrin glue, normal saline, hydrogel plugs) into the biopsy tract have been studied for their ability to decrease the risk of pneumothorax [3,4,5]. The use of an autologous blood patch is perhaps the most widely known technique, but it has shown varying results [6].

Previously, we evaluated the effect of needle-tract bleeding (NTB) on pneumothorax using post-biopsy CT images from a limited population for the first time [7].

This study aimed at determining the predictive variables for the development of NTB in a large patient population. We also evaluated whether NTB can function as a blood patch and prevent pneumothorax development and the resultant chest tube placement.

## Materials and Methods

### Patients

The protocol of this retrospective study was approved by the institutional review board. Written informed consent was not required because of the retrospective nature of the study.

From January 2001 to October 2006, 872 consecutive patients underwent CT-CNB. We excluded patients who had refractory coagulopathy (INR ≥ 1.5) or thrombocyte counts less than 75,000, lesions with a suspected vascular origin, severe respiratory disease (e.g., pulmonary arterial hypertension, or interstitial pulmonary disease), and those who refused to undergo the procedure. The four hundred biopsies in which the needle did not cross the aerated lung parenchyma were also excluded. Twenty-one cases of mediastinal lesions were excluded as well because they did not require a needle path to traverse through the aerated lungs. Thirty-eight patients who underwent only fine-needle aspiration biopsies were also excluded. The records of the remaining 416 patients who underwent biopsies with a non-coaxial cutting needle (338 men and 78 women; age range, 18–89 years; average age, 59.3 years) were evaluated. The predictive variables of pneumothorax and chest tube placement after CT-CNB of our cohort were reported in a previous study [8].

### Technique

All procedures were performed percutaneously under CT (Somatom Emotion; Siemens Healthcare, Forchheim, Germany) guidance by junior residents with a mean experience of five-biopsies and senior residents with a mean experience of 25 biopsies under the supervision of a chest radiologist with 20 years’ experience (a mean experience of 250 biopsies), who also performed some biopsies.

Details of the biopsy procedure were described in a previous report [8]. The patients were placed in the supine, prone, or lateral positions, and CT scans were obtained to target the lesion. Localization was performed using CT images with laser lighting and skin markers. The biopsy was routinely performed without any premedication or fasting. All patients received local anesthesia with subcutaneous injections of prilocaine hydrochlorate (Citanest 2%, AstraZeneca, Sweden).

After administration of the local anesthetic, the cutting needle was advanced directly in a stepwise fashion to obtain the specimen. Specimen acquisition was repeated until the radiologist considered the specimens to be sufficient, because of the absence of a pathologist on site. Cutting-needle biopsies were performed using an 18-gauge non-coaxial system (Meditech Inc., Watertown, MA 02172, USA).

Immediately after the procedure, a CT scan was obtained to determine the presence of pneumothorax. Patients were placed on a stretcher with the puncture site-down and were followed up for two hours. The patients were instructed to breathe calmly and abstain from talking, coughing, and forced breathing.

Chest radiographs were obtained at the end of the two-hour period. If pneumothorax was not present, the patient was sent home with instructions. If pneumothorax was detected on the control chest radiographs, the patient received oxygen via a facial mask for two hours if he/she was asymptomatic. Placement of a chest catheter or tube was considered if the patient became symptomatic or if the pneumothorax was progressive.

### Data collection and study outcomes

**Figure 1:**
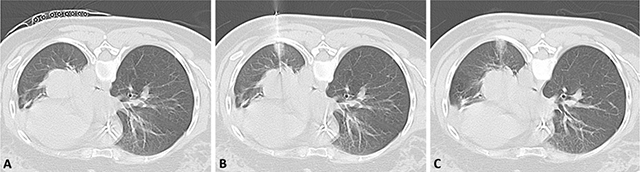
Needle-tract bleeding: **(A)** localization of needle entry; **(B)** during the procedure, needle is in the lesion; **(C)** after the withdrawal of the needle, bleeding is seen along the needle trajectory as ground-glass attenuation.

A linear increased attenuation extending from the pleural surface to the pulmonary lesion on intraprocedural CT images was accepted as a sign of NTB (Figure 1). Information regarding the patient, lesion, and procedure-related variables was collected to determine the risk factors for NTB, and the possible effect of NTB on pneumothorax development. The patient-related risk factors were age, gender, patient position (supine, prone, or lateral decubitus) during the procedure, and pulmonary emphysema around the lesion; the lesion-related risk factors were size, location within the lung (upper, middle, or lower lung regions), and contour characteristics on CT (smooth, blurred, or spiculated); and the procedure-related risk factors were the presence of atelectasis, pleural tags and fissures in the needle tract, length of the aerated lung parenchyma crossed by the needle, needle entry angle, number of pleural punctures, experience level of the operator, and duration of the procedure.

The distance from the pulmonary lesion to the pleura was estimated as the shortest distance on axial CT scans. Depth was considered to be 0 cm in lesions directly in contact with the pleura. The needle entry angle was the acute angle between the needle and the pleural surface. The lung was arbitrarily divided into three equal zones, i.e., upper, middle, and lower. The duration of the procedure was defined as the interval between the first CT image obtained to determine pleural passing and the first CT image obtained to check for the presence of pneumothorax. Emphysema around the lesion was scored as grade 1, 2, or 3 using the Goddard classification [9], which is a visual scale in which the areas showing vascular disruption and low attenuation values are scored for slices including the lesion. Emphysema around the lesion was considered grade 1 for ≤25%, grade 2 for 25%–50%, and grade 3 for >50%.

### Statistical analysis

All statistical comparisons related to the risk factors for pneumothorax and chest tube placement were performed using the software Statistical Package for the Social Sciences (SPSS, version 23.0; SPSS, Chicago, IL). Receiver operating characteristic (ROC) analysis was performed using an analysis program (MedCalc for Windows, Ostend, Belgium). Descriptive statistics were presented as the mean ± standard deviation for continuous variables and counts with a percentage for categorical variables. Gender was compared using Pearson’s chi-square test, and the Student t-test was used for assessing patient age. The ROC analysis was used to assess the optimal cutoff value for numerical variables such as age, lesion size, length of the aerated lung parenchyma crossed by the needle, needle entry angle, number of pleural punctures, and procedure duration (minimal false-negative and false-positive results) for the risk of NTB.

The rates of pneumothorax and chest tube placement were evaluated with stepwise regression analysis. A total of 421 lesions were divided into four groups depending on the occurrence of pneumothorax and NTB. Cases with pneumothoraces were divided into two groups according to the need for chest tube placement. The variables were compared between the two groups using univariate analysis with a two-sided Student’s t-test for numeric values and the chi-square test or Fisher’s exact test for categorical values. Subsequently, the variables that were found to be significantly different on univariate analyses were subjected to multivariate logistic regression analysis to determine the independent risk factors for the NTB and the possible effect of NTB on the occurrence of pneumothorax and consequent of chest tube placement. An odds ratio (OR) of >1.00 indicated a higher risk for the occurrence of NTB or the effect of the NTB on pneumothorax and chest tube placement. A p value of <0.05 was considered to be statistically significant.

## Results

The study included 421 needle biopsies performed in 416 patients (338 men and 78 women). Their ages ranged between 18 and 89 years (mean, 59.3 years). The biopsy was repeated three times in one patient and twice in three patients. Bleeding along the needle tract after CT-CNB was noted in 142 of the 421 procedures (33.7%).

On univariate analyses, the significant risk factors for NTB were identified as lesion size smaller than 3 cm (p < 0.001), greater lesion depth (p < 0.001), higher number of pleural passes (p = 0.008), and a needle entry angle < 75° (p = 0.007). Multivariate analysis showed that the significant independent risk factors were lesion size smaller than 3 cm (OR, 1.53; 95% CI, 1.31–1.94; p = 0.011) and greater lesion depth (OR, 1.90; 95% CI, 1.52–2.21; p = 0.002) (Tables 1, 2, 3).

**Table 1:** Correlation of the needle-tract bleeding with the lesion-related variables.

Variables	NTB Rate (%)	p-value

*Size(cm)*		**p = 0.011**

<3	68/170 (40%)	
≥3	74/251 (29%)	
*Localization*		p = 0.532

Upper	82/248 (33%)	
Middle	9/21 (42%)	
Lower	51/152 (34%)	
*Contour Characteristics*		p = 0.660

Smooth	30/101 (30%)	
Blurred	68/206 (33%)	
Spiculated	44/114 (38%)	

NTB, needle-tract bleeding.

**Table 2:** Correlation of the needle-tract bleeding with the procedure-related variables.

Variables	NTB Rate (%)	p-value

*Entry angle (°)*		p = 0.325

<75	59/150 (39%)	
≥75	83/271 (30%)	
*No of Pleu. Puncture*		p = 0.470

≤2	115/355 (32%)	
>2	27/66 (40%)	
*Time (minute)*		p = 0.692

<2.5	92/265 (35%)	
≥2.5	50/156 (32%)	
*Depth (cm)*		**p = 0.002**

0.5–3	38/158 (24%)	
>3	104/263 (40%)	
*Operator*		p = 0.842

Junior resident	33/89 (37%)	
Senior resident	69/228 (30%)	
Chest radiologist	40/104 (38%)	
*Others*		p = 0.105

Presence of atelectasis	2/12 (16%)	
Presence of fissure	10/22 (45%)	
Presence of pleu. Tag	3/12 (25%)	

NTB, needle-tract bleeding; Pleu. Puncture, pleural puncture; No, number.

**Table 3:** Correlation of the needle-tract bleeding with the patient-related variables.

Variables	NTB Rate (%)	p-value

*Age (y)*		p = 0.441

<60	78/207 (38%)	
≥60	64/214 (30%)	
*Gender*		p = 0.648

Male	116/337 (34%)	
Female	26/84 (31%)	
*Patient position*		p = 0.380

Supine	43/128 (33%)	
Prone	80/247 (32%)	
Lateral decubitus	19/46 (41%)	
*Severity of emphysema*		p = 0.218

0	95/285 (33%)	
1	31/80 (38%)	
2	13/34 (38%)	
3	3/22 (13%)	

NTB, needle-tract bleeding.

We observed only two cases (2/12) of NTB in the atelectatic needle trajectory. There was no significant difference in the bleeding rate when the needle passed through the fissure, atelectasis, or the pleural tag (p = 0.105) (Table 2). Contour characteristics of lesion (p = 0.660), patient position (p = 0.380), gender (p = 0.648), age (p = 0.441), severity of pulmonary emphysema around the lesion (p = 0.218), and location of the lesion (p = 0.532) were not associated with an increased risk of NTB (Tables 1, 2, 3).

Pneumothorax occurred in 115 of the 421 (27.3%) biopsies. Chest tube placement was required in 28 of these 115 (24.3%) cases. NTB was seen in 142 of the 421 (33.7%) biopsies and pneumothorax developed in 32 out of these 142 (22.5%) cases versus 83 of the 279 (29.7%) cases in which there was no NTB (p < 0.001). Out of the 32 cases showing both NTB and pneumothorax, 11 (34%) required chest tube placement. Among the 83 cases in which pneumothorax developed without NTB, 17 (20.4%) required chest tube placement (p = 0.092).

NTB was noted in 95 of the 285 (33.3%) cases without emphysema around the lesion, 31 of the 80 (38%) cases with grade 1 emphysema, 13 of the 34 (38%) cases with grade 2 emphysema, and three of the 22 (13%) cases with grade 3 emphysema. The differences between the groups were not statistically significant (p = 0.125). Twelve out of the 95 procedures with NTB and no emphysema had pneumothorax (12.6%) versus 44 of the 190 (23.1%) cases that did not show NTB (p < 0.001).

In cases with emphysema, pneumothorax occurred in 39 out of the 89 (44%) that did not show NTB versus 20 out of the 47 (42%) that showed NTB (p = 0.831) (Table 4).

**Table 4:** Correlation of pneumothorax with needle-tract bleeding and emphysema.

	With Emphysema				Without Emphysema			

	NTB	No NTB	Total (%)	*p-value*	NTB	No NTB	Total (%)	*p-value*

**Pneumothorax**				p = 0.831				**p < 0.001**
Not Seen	27	50	77		83	146	229	
Seen (%)	20 (42%)	39 (44%)	59 (43%)		12 (12%)	44 (32%)	56 (19%)	
Total	47	89	136		95	190	285	

NTB, needle tract bleeding.

## Discussion

Intrapulmonary bleeding was described by McCartney et al. [10] as a ‘bloom’ occurring after percutaneous lung biopsy. The risk of NTB associated with CT-CNB has been reported to range from 4% to 27% [7]. However, it is believed that this complication is under-reported [11]. In fact, at least to some extent, every percutaneous thoracic biopsy is related to some degree of pulmonary hemorrhage, which manifests as a focally increased attenuation in the needle track or in the perilesional lung tissue [12,13,14].

NTB was relatively common in our study (33%) in comparison with the incidence in previous studies (15%–26%). This form of bleeding was not a causative factor for any interruption of the biopsy procedure in our patients. Hemoptysis was extremely rare with the NTB, regardless of the amount of hemorrhage.

In this study, the procedure duration, localization of the lesion, needle entry angle, lesion contour, gender, presence of fissure or pleural tag in the needle path, and position of the patient did not influence the development of the NTB. A needle track longer than 3 cm within the lung parenchyma was significantly correlated with the occurrence of NTB. This correlation could partly be explained by the greater numbers of needles crossing the intrapulmonary small vessels in a longer intrapulmonary biopsy path as well as the larger diameter of the pulmonary vessels encountered as the needle comes closer to the hilar region.

Our data confirmed the results obtained by Yeow et al. [14] and Laurent et al. [15], who also found that smaller lesion size could be related to an increased risk of NTB. They hypothesized that for the smaller lesions, biopsies were technically more difficult and were associated with a higher number of needle path corrections, which might lead to a higher number of complications. Moreover, the transfissural needle trajectory was strongly correlated with both NTB and pneumothorax in the previous studies. However, in our series, the association of a transfissural needle path with the risk of NTB did not achieve statistical significance, probably because of the small number of procedures performed through a transfissural needle path.

The number of pleural punctures, as risk factors for pneumothorax and NTB development, has been fiercely debated in the literature. Some previous works have not shown any association between the number of pleural punctures and the incidence of pneumothorax [15,16], while some authors have found that multiple punctures could be associated with increased chances of pneumothorax. Ohno et al. [17] revealed that the incidence of pneumothorax was 18% after the first pleural puncture but significantly increased to 53% and 73% in the second and third pleural passes, respectively. In the current study, no association was found between the number of pleural passes and the rate of pneumothorax and NTB. It is unclear if this is a true relationship or an incidental finding of this retrospective study. Probably, the explanation for this is that when a pneumothorax occurred on the first pleural puncture, the procedure was terminated.

In the current study, we included all routine procedures that were performed by an experienced chest radiologist as well as the biopsies performed by senior and junior residents. When we evaluated the bleeding rate and the operator experience level, NTB was noted in 33 of the 89 (37%) procedures performed by junior residents, 69 out of the 228 (30%) procedures performed by senior residents, and 40 of the 104 (38%) procedures performed by the chest radiologist. In univariate analysis, differences between the operators were not significant.

NTB may have a similar effect as the blood-patch technique used to prevent pneumothorax. The blood-patch method is a popular technique in which the biopsy track is sealed with an autologous blood clot to prevent or reduce post-biopsy pneumothorax. The results of this technique are promising, and it has been reported to show a success rate of 85% in some retrospective studies [18,19,20]. Fibrin glue was shown to be an effective sealing agent for percutaneous lung biopsies [21]. However, the available fibrin sealants are not widely used due to the longer preparation time for these sealant agents, concerns regarding the risk of the infection, and the cost of the treatment.

The previous studies were performed mostly using agglutination materials within the biopsy track by causing a local reaction in the lung tissue. Our study suggests that the preventive effect of NTB against the development of pneumothorax could be at least as effective as agglutination techniques. Moreover, the adverse local tissue reaction that could potentially be induced by synthetic agglutination materials may be avoided by utilizing NTB as a natural protective factor.

To our knowledge, this was the first study revealing a correlation between NTB and pneumothorax in CT-CNBs in patients without emphysema around the lesion. NTB had a preventive effect against pneumothorax development in the absence of emphysema. This result remained significant even after adjustment for the lesion size and the lesion depth by multiple regression analysis. We speculated that this correlation might be related to the reduced ventilation as a result of the presence of blood products in the alveolar space. The patch-like alteration in the respiration may reduce the airflow from the airspace to the pleural space.

Unexpectedly, our previous work [7] with a small number of patients has shown a decreased rate of pneumothorax in the presence of NTB in patients having emphysema with borderline statistical significance. Some substantial differences exist between the studies. The present study enrolled 416 patients, while that of the previous study involved 284 patients. The number of patients with NTB for the present study was 142 compared with 53 for our previous study. Most importantly, our previous work has not taken potentially confounding variables associated with a CT-CNB such as patient position, contour characteristics of the lesion, needle entry angle, and procedure duration into account, which is at the heart of our current analysis.

The major limitations of our study were related to the non-use of coaxial needles and the retrospective nature of the study. However, the study population was homogeneous and suitable for the appropriate statistical analyses with regard to the predictive variables. Another limitation of our study was the lack of a prospective validation step and the absence of long-term follow-up data for patients who developed pulmonary hemorrhage during CT-CNB. Test for predictive variables, like those performed in our study, could be associated with an inflated type I error. In our series, the severity of emphysema is evaluated by Goddard classification which is a visual scale that area of vascular disruption and low attenuation value were scored for each lung field. The emphysema score used by the radiologists in our study was a non-standardized index based on the individual judgement of the radiologist regarding the extent of emphysema in the lobe containing the lesion. Consequently, there might be both interobserver and intraobserver variations in scoring emphysema according to the Goddard classification. Further studies are needed to determine whether the presence of emphysema measured by quantitative standardized criteria in the lung region containing the lesion represents a factor for the development of pneumothorax.

In conclusion, NTB occurs more frequently in smaller and deeply seated lung lesions in CT-guided percutaneous lung biopsies. NTB may have a protective effect against pneumothorax, but not against chest tube placement, in the absence of emphysema around the lesion.

## Figures and Tables

**Table:** 

**NTB**	Needle-tract bleeding
**CT**	Computed tomography
**CNB**	Core needle biopsy
**IRB**	Institutional review board
**G**	Gauge
**ROC**	Receiver operating characteristic
**OR**	Odds ratio

## References

[B1] Heerink, WJ, de Bock, GH, de Jonge, GJ, Groen, HJ, Vliegenthart, R and Oudkerk, M. Complication rates of CT-guided transthoracic lung biopsy: Meta-analysis. Eur Radiol. 2017; 27: 138–48. DOI: 10.1007/s00330-016-4357-8 27108299 PMC5127875

[B2] Tsai, IC, Tsai, WL, Chen, MC, et al. CT-guided core biopsy of lung lesions: A primer. AJR Am J Roentgenol. 2009; 193: 1228–35. DOI: 10.2214/AJR.08.2113 19843735

[B3] Zaetta, JM, Licht, MO, Fisher, JS and Avelar, RL. A lung biopsy tract plug for reduction of post-biopsy pneumothorax and other complications: Results of a prospective, multicenter, randomized, controlled clinical study. J Vasc Interv Radiol. 2010; 21: 1235–43. DOI: 10.1016/j.jvir.2010.04.021 20656223

[B4] Billich, C, Muche, R, Brenner, G, et al. CT-guided lung biopsy: Incidence of pneumothorax after instillation of NaCl into the biopsy track. Eur Radiol. 2008; 18: 1146–52. DOI: 10.1007/s00330-008-0872-6 18270713

[B5] Ahrar, JU, Gupta, S, Ensor, JE, et al. Efficacy of a self-expanding tract sealant device in the reduction of pneumothorax and chest tube placement rates after percutaneous lung biopsy: A matched controlled study using propensity score analysis. Cardiovasc Intervent Radiol. 2017; 40: 270–6. DOI: 10.1007/s00270-016-1489-9 27826786 PMC5215969

[B6] Wagner, JM, Hinshaw, JL, Lubner, MG, et al. CT-guided lung biopsies: Pleural blood patching reduces the rate of chest tube placement for post-biopsy pneumothorax. AJR Am J Roentgenol. 2011; 197: 783–8. DOI: 10.2214/AJR.10.6324 21940564

[B7] Topal, U and Berkman, YM. Effect of needle tract bleeding on occurrence of pneumothorax after transthoracic needle biopsy. Eur J Radiol. 2005; 53: 495–9. DOI: 10.1016/j.ejrad.2004.05.008 16021686

[B8] Ozturk, K, Soylu, E, Gokalp, G and Topal, U. Risk factors of pneumothorax and chest tube placement after computed tomography-guided core needle biopsy of lung lesions: A single-centre experience with 822 biopsies. Pol J Radiol. 2018; 83: 407–14. DOI: 10.5114/pjr.2018.79205 PMC6334126 30655918

[B9] Goddard, PR, Nicholson, EU, Laszlo, G and Watt, I. Computed tomography in pulmonary emphysema. Clin Radiol. 1982; 33: 379–87. DOI: 10.1016/S0009-9260(82)80301-2 7083738

[B10] McCartney, RL. Hemorrhage following percutaneous lung biopsy. Radiology. 1974; 112: 305–7. DOI: 10.1148/112.2.305 4835025

[B11] Tai, R, Dunne, RM, Trotman-Dickenson, B, et al. Frequency and severity of pulmonary hemorrhage in patients undergoing percutaneous CT-guided transthoracic lung biopsy: Single-institution experience of 1175 Cases. Radiology. 2015; 279: 287–96. DOI: 10.1148/radiol.2015150381 26479161

[B12] De Filippo, M, Saba, L, Silva, M, et al. CT-guided biopsy of pulmonary nodules: Is pulmonary hemorrhage a complication or an advantage? Diagn Interv Radiol. 2014; 20: 421 DOI: 10.5152/dir.2014.14019 25163758 PMC4463325

[B13] Khan, MF, Straub, R, Moghaddam, SR, et al. Variables affecting the risk of pneumothorax and intrapulmonal hemorrhage in CT-guided transthoracic biopsy. Eur Radiol. 2008; 18: 1356–63. DOI: 10.1007/s00330-008-0893-1 18351356

[B14] Yeow, KM, Su, IH, Pan, KT, et al. Risk factors of pneumothorax and bleeding: Multivariate analysis of 660 CT-guided coaxial cutting needle lung biopsies. Chest. 2004; 126: 748–54. DOI: 10.1378/chest.126.3.748 15364752

[B15] Laurent, F, Latrabe, V, Vergier, B, Montaudon, M, Vernejoux, JM and Dubrez, J. CT-guided transthoracic needle biopsy of pulmonary nodules smaller than 20 mm: Results with automated 20-gauge coaxial cutting needle. Clin Radiol. 2000; 55: 281–7. DOI: 10.1053/crad.1999.0368 10767187

[B16] Anderson, JM, Murchison, J and Patel, D. CT-guided lung biopsy: Factors influencing diagnostic yield and complication rate. Clin Radiol. 2003; 58: 791–7. DOI: 10.1016/S0009-9260(03)00221-6 14521889

[B17] Ohno, Y, Hatabu, H, Takenaka, D, et al. CT-guided transthoracic needle aspiration biopsy of small (≤20 mm) solitary pulmonary nodules. AJR Am J Roentgenol. 2003; 180: 1665–9. DOI: 10.2214/ajr.180.6.1801665 12760939

[B18] Clayton, JD, Elicker, BM, Ordovas, KG, Kohi, MP, Nguyen, J and Naeger, DM. Nonclotted blood patch technique reduces pneumothorax and chest tube placement rates after percutaneous lung biopsies. J Thorac Dis. 2016; 31: 243–6. DOI: 10.1097/RTI.0000000000000215 27105052

[B19] Lang, EK, Ghavami, R, Schreiner, VC, Archibald, S and Ramirez, J. Autologous blood clot seal to prevent pneumothorax at CT-guided lung biopsy. Radiology. 2000; 216: 93–6. DOI: 10.1148/radiology.216.1.r00jl3293 10887232

[B20] Malone, LJ, Stanfill, RM, Wang, H, Fahey, KM and Bertino, RE. Effect of intraparenchymal blood patch on rates of pneumothorax and pneumothorax requiring chest tube placement after percutaneous lung biopsy. AJR Am J Roentgenol. 2013; 200: 1238–43. DOI: 10.2214/AJR.12.8980 23701059

[B21] Petsas, T, Siamblis, D, Giannakenas, C, et al. Fibrin glue for sealing the needle track in fine-needle percutaneous lung biopsy using a coaxial system: Part II—clinical study. Cardiovasc Intervent Radiol. 1995; 18: 378–82. DOI: 10.1007/BF00338305 8591624

